# Supplementary material for: SyNDI: synchronous network data integration framework
Source: BMC Bioinformatics. 2018 Nov 6;19:403. doi: 10.1186/s12859-018-2426-5 (PMC6219086; doi:10.1186/s12859-018-2426-5)
Supplement: Supplementary file 5 — Files of the example presented in Additional file 4. This additional file is a zip package that contains all files used in Additional file 4. Detailed descriptions of each of these files are presented in a README file included in this zip package. (GZ 30411 kb) [file 12859_2018_2426_MOESM5_ESM.gz › SyNDITools/Meme2Fimo/meme_4.9.0_4.tar.gz!/meme_4.9.0/doc/clustalw-format.html]

Clustal W file format


# Clustal W file format

Various programs in the MEME Suite allow as input a file containing a
multiple alignment of protein or DNA sequences. These input files
must be in CLUSTAL W format (usually identified with the suffix
".aln").

The format is very simple:

1. The first line in the file must start with the words "CLUSTAL W" or "CLUSTALW".
   Other information in the first line is ignored.
2. One or more empty lines.
3. One or more blocks of sequence data. Each block consists of:

- One line for each sequence in the alignment. Each line consists of:

1. the sequence name
2. white space
3. up to 60 sequence symbols.
4. optional - white space followed by a cumulative count of residues for the sequences

- A line showing the degree of conservation for the columns
  of the alignment in this block.
- One or more empty lines.

Some rules about representing sequences:

- Case doesn't matter.
- Sequence symbols should be from a valid
  alphabet.- Gaps are represented using hyphens ("-").
  - The characters used to represent the degree of conservation are

    ```
             *  -- all residues or nucleotides in that column are identical
             :  -- conserved substitutions have been observed
             .  -- semi-conserved substitutions have been observed
                -- no match.
    ```

Here is an example of a multiple alignment in CLUSTAL W format:

```
      CLUSTAL W (1.82) multiple sequence alignment


      FOSB_MOUSE      MFQAFPGDYDSGSRCSSSPSAESQYLSSVDSFGSPPTAAASQECAGLGEMPGSFVPTVTA 60
      FOSB_HUMAN      MFQAFPGDYDSGSRCSSSPSAESQYLSSVDSFGSPPTAAASQECAGLGEMPGSFVPTVTA 60
                      ************************************************************

      FOSB_MOUSE      ITTSQDLQWLVQPTLISSMAQSQGQPLASQPPAVDPYDMPGTSYSTPGLSAYSTGGASGS 120
      FOSB_HUMAN      ITTSQDLQWLVQPTLISSMAQSQGQPLASQPPVVDPYDMPGTSYSTPGMSGYSSGGASGS 120
                      ********************************.***************:*.**:******

      FOSB_MOUSE      GGPSTSTTTSGPVSARPARARPRRPREETLTPEEEEKRRVRRERNKLAAAKCRNRRRELT 180
      FOSB_HUMAN      GGPSTSGTTSGPGPARPARARPRRPREETLTPEEEEKRRVRRERNKLAAAKCRNRRRELT 180
                      ****** ***** .**********************************************

      FOSB_MOUSE      DRLQAETDQLEEEKAELESEIAELQKEKERLEFVLVAHKPGCKIPYEEGPGPGPLAEVRD 240
      FOSB_HUMAN      DRLQAETDQLEEEKAELESEIAELQKEKERLEFVLVAHKPGCKIPYEEGPGPGPLAEVRD 240
                      ************************************************************

      FOSB_MOUSE      LPGSTSAKEDGFGWLLPPPPPPPLPFQSSRDAPPNLTASLFTHSEVQVLGDPFPVVSPSY 300
      FOSB_HUMAN      LPGSAPAKEDGFSWLLPPPPPPPLPFQTSQDAPPNLTASLFTHSEVQVLGDPFPVVNPSY 300
                      ****:.******.**************:*:**************************.***

      FOSB_MOUSE      TSSFVLTCPEVSAFAGAQRTSGSEQPSDPLNSPSLLAL 338
      FOSB_HUMAN      TSSFVLTCPEVSAFAGAQRTSGSDQPSDPLNSPSLLAL 338
                      ***********************:**************
```

Further information about the CLUSTAL format can be found
here
